# Systemic chemotherapy induces microsatellite instability in the peripheral blood mononuclear cells of breast cancer patients

**DOI:** 10.1186/bcr950

**Published:** 2004-11-04

**Authors:** Fernando LA Fonseca, Aleksandra VL Sant Ana, Israel Bendit, Vitor Arias, Luciano J Costa, Aparecida A Pinhal, Auro del Giglio

**Affiliations:** 1 ABC Foundation School of Medicine, Hematology and Oncology, Santo Andre, São Paulo, Brazil; 2 Faculdade de Medicina da Universidade de São Paulo, Hematology Post Graduate Section, São Paulo, Brazil; 3 Hemocentro de São Paulo, Tumor Biology, São Paulo, Brazil; 4 Instituto Adolfo Lutz, Anatomic Pathology, São Paulo, Brazil; 5 Faculdade de Medicina da Universidade de São Paulo, Pathology Post Graduate Section, São Paulo, Brazil; 6 ABC Foundation School of Medicine, Biochemistry, São Paulo, Brazil

**Keywords:** cyclophosphamide, microsatellite repeats, neoplasms, proliferating cell nuclear antigen, second primary

## Abstract

**Introduction:**

Systemic chemotherapy is an important part of treatment for breast cancer. We conducted the present study to evaluate whether systemic chemotherapy could produce microsatellite instability (MSI) in the peripheral blood mononuclear cell fraction of breast cancer patients.

**Methods:**

We studied 119 sequential blood samples from 30 previously untreated breast cancer patients before, during and after chemotherapy. For comparison, we also evaluated 20 women who had no relevant medical history (control group).

**Results:**

In 27 out of 30 patients we observed MSI in at least one sample, and six patients had loss of heterozygosity. We found a significant correlation between the number of MSI events per sample and chemotherapy with alkylating agents (*P *< 0.0001). We also observed an inverse correlation between the percentage of cells positive for hMSH2 and the number of MSI events per sample (*P *= 0.00019) and use of alkylating agents (*P *= 0.019).

**Conclusion:**

We conclude that systemic chemotherapy may induce MSI and loss of heterozygosity in peripheral blood mononuclear cells from breast cancer patients receiving alkylating agents, possibly mediated by a chemotherapy-induced decrease in the expression of hMSH2. These effects may be related to the generation of secondary leukaemia in some patients, and may also intensify the genetic instability of tumours and increase resistance to treatment.

## Introduction

Systemic chemotherapy is an important part of treatment for breast cancer in both the adjuvant and palliative settings. Despite the consistent improvement in overall survival afforded by systemic adjuvant chemotherapy, about 1% of patients develop secondary leukaemia and/or myelodysplasia, probably as a consequence of the genotoxic effects of this type of treatment [1]. Deficiencies in the DNA mismatch repair system leading to microsatellite instability (MSI) may produce a syndrome of familial predisposition to colon, endometrial and upper gastrointestinal cancers, known as hereditary nonpolyposis colon cancer [2]. The DNA mismatch repair system depends on the coordinated interplay of several proteins encoded by various genes, including hMLH1, hMSH2, hPMS1 and hPMS2 [2]. Several groups have reported a significant association between MSI and treatment-related secondary acute myeloid leukaemia (AML) and myelodysplasia [3-5]. In some studies MSI in treatment-related secondary AML/myelodysplasia cases was accompanied by hMLH1 hypermethylation [3] and MSH2 polymorphisms [6].

In order to evaluate whether systemic chemotherapy could produce MSI in normal peripheral blood mononuclear cells (PBMCs), we analyzed PBMCs from breast cancer patients collected before, during and after systemic chemotherapy.

## Methods

### Collection and preparation of samples

This protocol was approved by our institutional review board. We obtained blood samples from 33 patients with histologically confirmed breast cancer after informed consent had been obtained. We had only the initial sample in three patients, and so we could not include them in the study. We therefore studied 119 sequential blood samples from 30 previously untreated breast cancer patients, collected at 3-month intervals before, during and after receiving systemic treatment (13 adjuvant, 12 neoadjuvant and 5 palliative). Three patients initially received hormones (two adjuvant and one palliative).

Chemotherapy combinations containing cyclophosphamide were classified as alkylating regimens (FAC [5-fluorouracil, doxorubicin and cyclophosphamide], AC [doxorubicin and cyclophosphamide], CMF [cyclophosphamide, methotrexate, and 5-fluorouracil]). We also studied PBMCs from 20 normal control women, who had no relevant previous medical history, by immunocytochemistry. Afterward, we collected peripheral blood from these 20 normal control women on two occasions with a 3-month interval to evaluate MSI using the TP53Alu and PCR15.1 markers (described below).

Each sample consisted of 20 ml venous blood, from which we separated the PBMCs by Ficoll gradient (Ficoll Hypaque; Organon Teknica^®^, Durham, NC, USA), yielding a final concentration of 1.0 × 10^6 ^cells/ml; we sent part of the sample for cytospin for immunocytochemical studies and part for DNA extraction. DNA was extracted from mononuclear fraction by the use of Trizol (Invitrogen, Carlsbad, CA, USA), in accordance with the manufacturer's instructions. For immunocytochemistry analysis, assay slides were prepared (described in detail elsewhere [7]).

### Microsatellite analysis

The sequences of all primers used for six microsatellite loci (BAT-26, BAT-40, MFD-28, MFD-41, TP53, PCR15.1, TP53ALU) and the PCR protocols used to study each of these markers were described previously [8]. Briefly, PCR products were denatured and subjected to electrophoresis in Gene Gel Clean 15/24 (Amershan Pharmacia Biotech AB, Uppsala, Sweden) for 90 min at 600 V and 8°C, and then silver stained using a Hoefer Automed Gel Stainer (Amershan Pharmacia Biotech AB). MSI was defined by the appearance of novel bands and loss of heterozygosity (LOH) whenever disappearance of previously visualized bands occurred, as described previously [8]. Three independent observers analyzed the results of each gel for LOH and MSI.

### Immunocytochemistry

For immunostaining for proliferating nuclear antigen (PCNA), hMLH1, hPMS1, hPMS2, hMSH2 and TP-53 proteins, we used an avidin–biotin–peroxidase complex and 3,3'-diaminobenzidine as chromogen. All antibodies were supplied by Santa Cruz Biotechnology (Santa Cruz, CA, USA), and for each clone we employed the following dilutions: PC-10 (anti-PCNA), 1:1000; C-20 (anti-hMLH1), 1:25; K-20 (anti-hPMS1), 1:25; C-20 (anti-hPMS2), 1:200; N-20 (anti-hMSH2), 1:200; and Pab1801 (anti-P53), 1:400. Endogenous peroxidase activity was blocked by incubation with H_2_O_2 _and washed in tap water. The cytospin slides were then treated with 7% skimmed milk for 60 min to block nonspecific protein binding. Each antibody was applied separately, and cytospins were incubated for 18 hours at 3°C. After 3 washes in phosphate-buffered saline, antigen-bound primary antibody was detected using a standard streptavidin–biotin complex (Strep AB Complex; Dako, Carpinteria, CA, USA). After brief washing, slides were incubated with diaminobenzidine and H_2_O_2 _for 10 min and then counterstained with haematoxylin, dehydrated in graded alcohols and cleared in xylene. Two independent observers scored 300 cells/slide as positive or negative according to the presence of nuclear staining for each of the aforementioned antibodies. Then, the results from these two observers were averaged to obtain a percentage of positive cells per sample.

### Statistical methods

We analyzed correlations between categorical variables using the χ^2 ^or Fisher's exact test. We used analysis of variance to study correlations between continuous and categorical variables, and simple regression to analyze correlations between continuous variables.

## Results

We studied 30 patients with median age 52 years (range 25–80 years). Twelve patients had stage II, 13 had stage III and five had stage IV breast cancer. We evaluated samples from 20 normal control women by immunocytochemistry for PCNA, hMLH1, hPMS2, hMSH2 and TP-53 (Table 1); the raw data for each of the included patients appear in Additional file 1.

**Figure 1:**
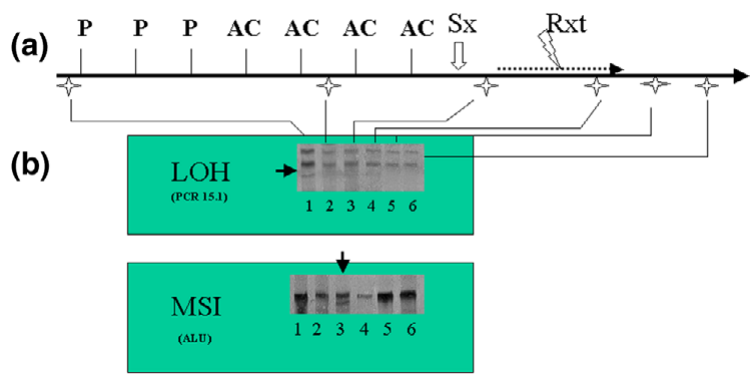
**Loss of heterozygosity (LOH) and microsatellite instability (MSI) occurrence. **(a) The clinical course of one of the studied patients who received three cycles of neoadjuvant paclitaxel (P) followed by four cycles of neoadjuvant doxorubicin with cyclophosphamide (AC) before surgery (Sx) and radiation therapy (Rxt). Each star below the straight line indicates a blood sample collection (time intervals between collections are not to scale). **(b) **Single-strand conformation polymorphism (SSCP) gels for two MSI markers PCR15.1 and ALU from the patient represented in panel a, showing the occurrence of LOH and MSI (black arrows). Each lane corresponds to one of the blood samples collected from this patient. Note that MSI disappears whereas LOH persists.

We observed MSI in 40 out of the 119 samples collected, and MSI was observed in 27 out of 30 patients in at least one sample. We observed LOH at the PCR15.1 locus in six patients. Whereas MSI was usually transient, disappearing in the next collected sample, LOH was persistent in all subsequent samples in five of the six observed cases (Fig. 1).

In order to ensure that our findings in the patients studied were not due to chance, we studied 20 normal blood donors (women who had no relevant medical history; see Additional file 2). In those women blood samples were collected twice, with a 3-month interval between samples. We studied both samples from each of these normal women for MSI using TP53ALU and PCR15.1 markers. In only one normal woman was any MSI identified (one additional band was detected in the PCR15-1 marker after 3 months). None of them had LOH (data not shown). Therefore, in only one blood sample out of 40 drawn from these 20 normal women was there any evidence of MSI, as compared with 28 out of 119 samples from the breast cancer patients studied using the same markers (*P *= 0.018).

**Figure 2:**
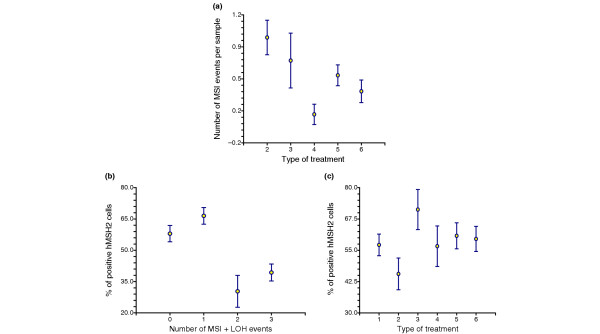
**Correlations between microsatellite instability (MSI) events, treatment, and hMSH2-positive cells.** Significant correlations between **(a) **number of MSI events and type of treatment received by patients during which samples were collected (2, alkylating chemotherapy; 3, nonalkylating chemotherapy; 4, radiation therapy; 5, hormone therapy; and 6, off treatment), **(b) **percentage of hMSH2-positive cells and number of MSI events (MSI and loss of heterozygosity [LOH]), and **(c) **percentage of hMSH2-positive cells and type of treatment received by patients during which samples were collected (1, before treatment; 2, alkylating chemotherapy; 3, nonalkylating chemotherapy; 4, radiation therapy; 5, hormone therapy; 6, off treatment). Values are expressed as means and standard errors (for *P *values see text).

We observed a significant correlation between the number of MSI events per sample and the use of chemotherapy (*P *= 0.005), especially chemotherapy containing alkylating agents (*P *< 0.0001; Fig. 2). We also observed an inverse correlation between the number of MSI events per sample and the percentage of cells positive for MSH2 by immunocytochemistry (*P *= 0.00019) and especially for the MFD41 marker (*P *= 0.000638). The percentage of cells positive for MSH2 by immunocytochemistry was inversely correlated with the use of chemotherapy regimens containing alkylating agents (*P *= 0.019). We found no significant correlations between the number of MSI events or the presence of LOH and the frequency of cells positive for TP53, hMLH1, hPMS1 and hPMS2.

In the present study, the percentage of cells positive for PCNA, as determined by immunocytochemistry, was directly correlated with use of radiation therapy (*P *= 0.046) and with the percentage of MSH2-positive cells (*P *= 0.000096), and inversely correlated with the presence of LOH (*P *= 0.043), but it was not correlated with the number of MSI events per sample.

## Discussion

Systemic chemotherapy administered to breast cancer patients induces secondary leukaemia and myelodysplasia in about 1% [1]. In our study, in 90% of patients MSI was noted in at least one of the samples. This finding does not appear due to chance because only one of the sequential blood samples drawn from 20 normal donor women exhibited MSI. Furthermore, we observed a significant and direct correlation between the number of MSI events per sample and the use of chemotherapy, especially with regimens containing alkylating agents. We believe that our data reflect the genotoxic effects of chemotherapy on normal cells because in only one normal control woman out of 20 was there evidence of MSI (after 3 months) and with only one of the two surveyed MSI markers. Furthermore, no LOH was observed within this cohort of normal women. Therefore, because MSI and LOH occurred at significantly greater frequency among patients with breast cancer, it is unlikely that our findings could be due to normal variations in MSI occurrence or to technical artifacts.

We also noted a significant, inverse correlation between the number of MSI events per sample and the expression of hMSH2 by immunocytochemistry. Because we noted fluctuation in hMSH2 levels throughout the treatment of several patients, it is possible that hMSH2 downregulation occurs at the post-transcriptional level and could be related to the use of chemotherapy based on alkylating agents. Interestingly, Watanabe and coworkers [9] described induction of MSI in ovarian tumours from patients studied before and after they received cisplatin-based chemotherapy, but in their study the cisplatin-induced MSI correlated with a significant decrease in hMLH1 expression.

We believe that the transient decrease in hMSH2 expression that we noted might have predisposed to an increased number of MSI events. In fact, Zhu and coworkers [10] also described abnormal hMSH-2 expression in about one-third of cases of AML, mainly in those with treatment-related secondary AML and in patients who were elderly.

PCNA is a 36 kDa nuclear protein that functions as a cofactor for δ-DNA polymerase, which is regulated in a cell cycle dependent manner. PCNA expression also increases when cells are actively engaged in DNA repair [11]. The correlation of PCNA with LOH but not with the number of MSI events found in the present study may indicate that PCNA may have greater expression in situations of more intense DNA damage that tend to be long-lasting. Interestingly, our group previously showed that PCNA over-expression was associated with resistance to chemotherapy AML and chronic lymphocytic leukaemia [12,13].

## Conclusion

We conclude that systemic chemotherapy may induce MSI and LOH in PBMCs from breast cancer patients receiving chemotherapy regimens, especially chemotherapy regimens containing alkylating agents. Furthermore, it is possible that these alterations are associated with a chemotherapy-induced decrease in the expression of hMSH2. These effects may be related to the generation of secondary leukaemia in some patients, and may also intensify the genetic instability of tumours and increase resistance to treatment.

## Abbreviations

AML = acute myeloid leukaemia; LOH = loss of heterozygosity; MSI = microsatellite instability; PBMC = peripheral blood mononuclear cell; PCNA = proliferating nuclear antigen; PCR = polymerase chain reaction.

## Competing interests

The author(s) declare that they have no competing interests.

## Supplementary Material

Additional File 1 An Excel file showing raw data for each patient studied. Click here for file

Additional File 2 A figure showing the results of testing in 20 normal women. The 20 normal women were tested for MSI using the PCR15.1 and TP53Alu markers. Each normal woman's sample corresponds to one letter (e.g. 'A' represents the first sample of the first control woman and 'A1' her second sample, collected 3 months latter). We noted only one case (S and S1) in which the second sample showed an extra band with the marker PCR15.1 (indicated by an arrow). Click here for file

## Figures and Tables

**Table 1:** Peripheral blood mononuclear cells positive for each of the studied markers by immunocytochemistry

Protein	Percentage
PCNA	15.93 ± 4.38
hMLH1	0.25 ± 1.11
hPMS1	0.6 ± 0.99
hPMS2	0 ± 0
hMSH2	72.7 ± 20.33
TP53	0 ± 0

Values are expressed as mean ± standard deviation (%).
