# Supplementary material for: Systemic chemotherapy induces microsatellite instability in the peripheral blood mononuclear cells of breast cancer patients
Source: Breast Cancer Res. 2004 Nov 4;7(1):R28–32. doi: 10.1186/bcr950 (PMC1064099; doi:10.1186/bcr950)
Supplement: Additional File 2 — A figure showing the results of testing in 20 normal women. The 20 normal women were tested for MSI using the PCR15.1 and TP53Alu markers. Each normal woman's sample corresponds to one letter (e.g. 'A' represents the first sample of the first control woman and 'A1' her second sample, collected 3 months latter). We noted only one case (S and S1) in which the second sample showed an extra band with the marker PCR15.1 (indicated by an arrow). [file bcr950-S2.ppt]

## Slide 1
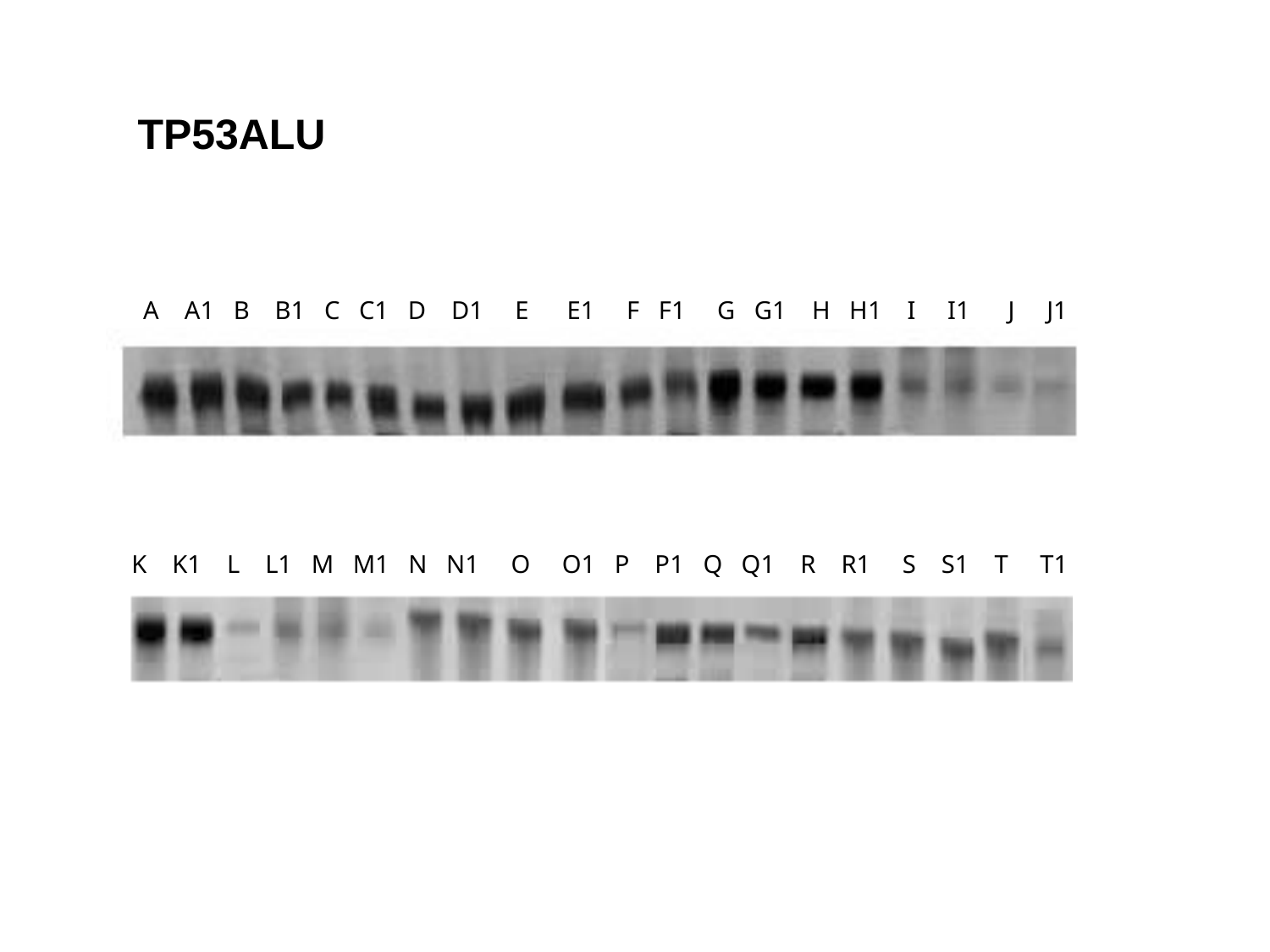

TP53ALU
A A1 B B1 C C1 D D1 E E1 F F1 G G1 H H1 I I1 J J1
K K1 L L1 M M1 N N1 O O1 P P1 Q Q1 R R1 S S1 T T1

## Slide 2
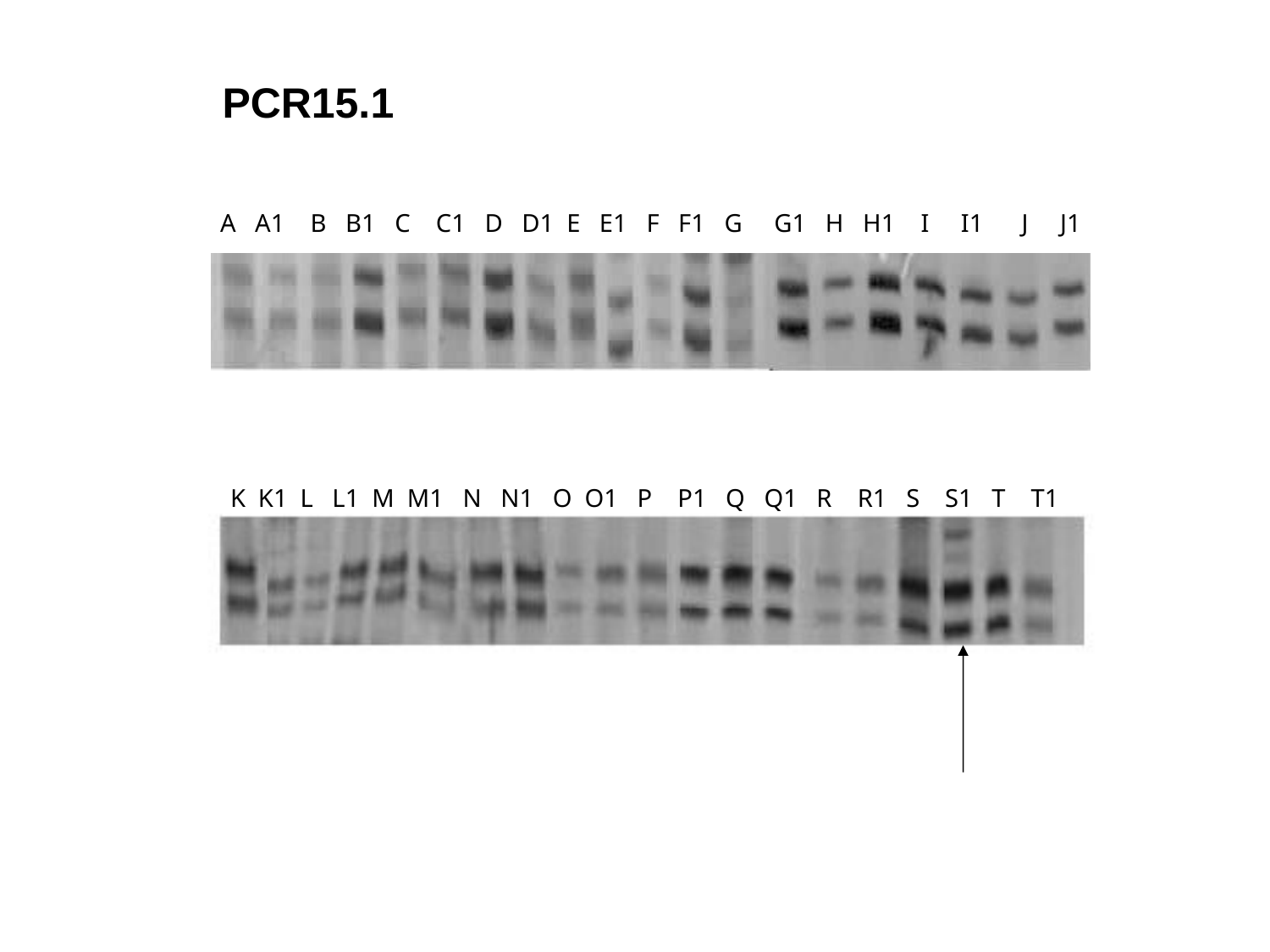

PCR15.1
A A1 B B1 C C1 D D1 E E1 F F1 G G1 H H1 I I1 J J1
 K K1 L L1 M M1 N N1 O O1 P P1 Q Q1 R R1 S S1 T T1
K K1 L L1 M M1 N N1 O O1 P P1 Q Q1 R R1 S S1 T T1

## Slide 3
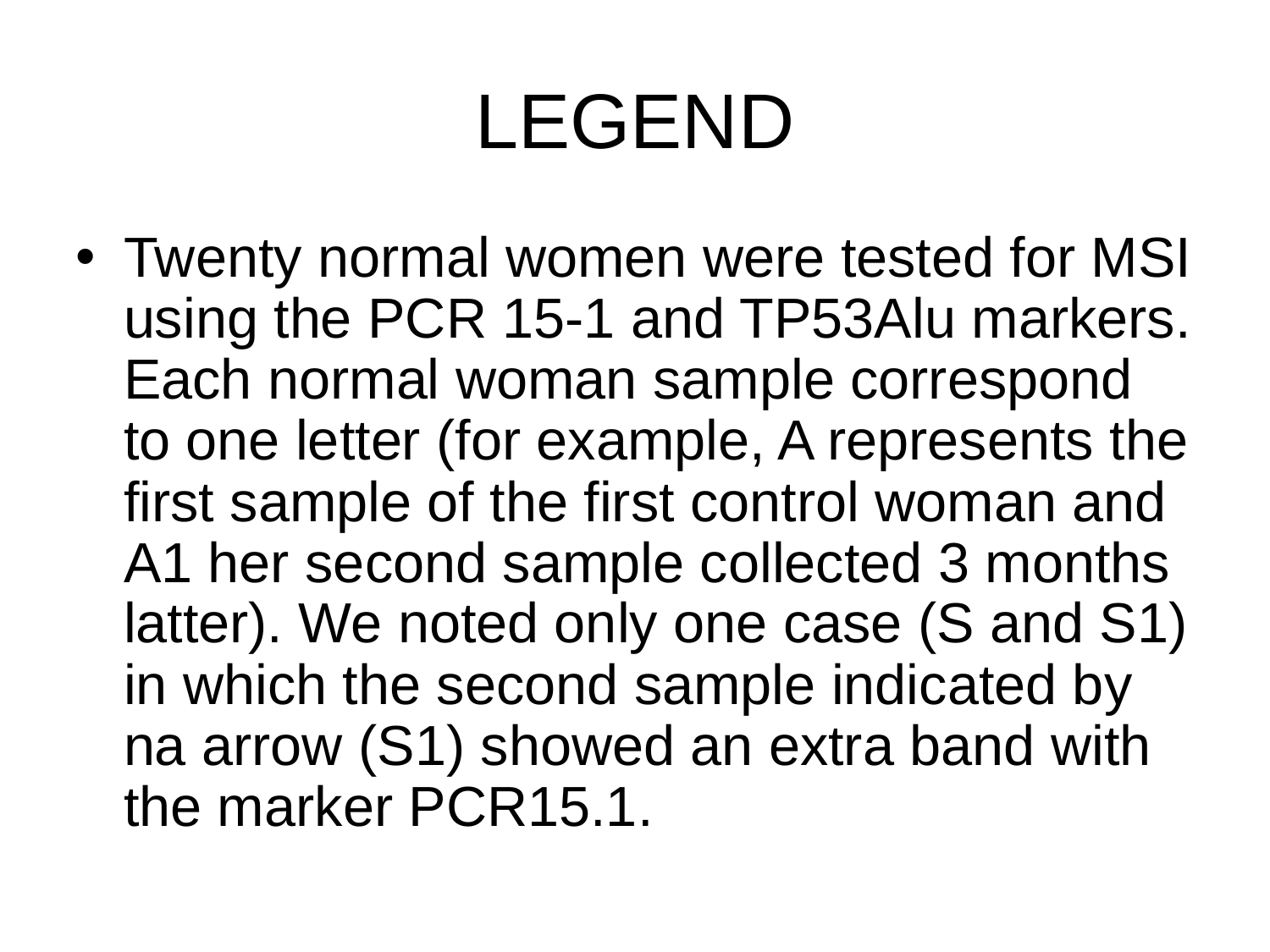

# LEGEND
Twenty normal women were tested for MSI using the PCR 15-1 and TP53Alu markers. Each normal woman sample correspond to one letter (for example, A represents the first sample of the first control woman and A1 her second sample collected 3 months latter). We noted only one case (S and S1) in which the second sample indicated by na arrow (S1) showed an extra band with the marker PCR15.1.
